# 

**Published:** 1879-06

**Authors:** 


### CONTENTS.

### COMMUNICATIONS.

Alveolar Abscess. *By M. F. Finley, D. D. S* .... 229
A New Method of Inserting an Artificial Crown upon a Root. *By H. A. Moyer.* 234

### PROCEEDINGS OF SOCIETIES.

Thirty-fifth Annual Meeting of the Mississippi Valley Association of Dental Surgeons .... 237
Academie De Medicine et Des Sciences, Paris .... 241
The Convention of the New England Dental Societies .... 245

### MISCELLANEOUS.

How We Judge Distances. *By C. H. Stowell, M. D* .... 246
Wheat Phosphates *By Elwood C. Lester, M. D., Penna* .... 248
The Metric System .... 250
Pathology of Tetanus .... 251
What Shall We Eat? .... 252
American Health Primers .... 253
The Little Helper. *By J. G. Harper, D. D. S* .... 255
The Formation of Character .... 256
Ether with Cod Liver Oil .... 257
A Fallacious Theory Exploded .... 258
A Pleasant Method of Administering Castor Oil .... 259
A Cause of Anæmia .... 259

### EDITORIAL.

Professional Education .... 260
Elephantine Dentals .... 262
Dental Vulcanite Co .... 264
The National Dental Association .... 266
Ho! For Niagara .... 266
Bibliographical .... 267
A New Dental College .... 267
Meeting .... 2[illegible]8
A New Clamp .... 269
A New Periodical .... 270
Another Dental Law .... 270

